# Supplementary material for: A statistical physics framework for optimal learning
Source: PNAS Nexus. 2026 May 26;5(6):pgag182. doi: 10.1093/pnasnexus/pgag182 (PMC13253564; doi:10.1093/pnasnexus/pgag182)
Supplement: pgag182_Supplementary_Data [file pgag182_supplementary_data.pdf]

Supporting Information

A statistical physics framework for optimal learning

Francesca Mignacco Francesco Mori

S1 Derivation of the learning dynamics

In this section, we derive the set of ordinary differential equations (ODEs) for the order parameters given the main text, that track the dynamics of online stochastic gradient descent (SGD). We consider the cost function

$$\mathcal{L}(\boldsymbol{w},\boldsymbol{v}|\boldsymbol{x},\boldsymbol{c}) = \ell\left(\frac{\boldsymbol{x}^\top \boldsymbol{w}_*}{\sqrt{N}},\frac{\boldsymbol{x}^\top \boldsymbol{w}}{\sqrt{N}},\frac{\boldsymbol{w}^\top \boldsymbol{w}}{N},\boldsymbol{v},\boldsymbol{c},z\right) + \tilde{g}\left(\frac{\boldsymbol{w}^\top \boldsymbol{w}}{N},\boldsymbol{v}\right) . \tag{S1}$$

The update rules for the network's parameters are

$$\boldsymbol{w}^{\mu+1} = \boldsymbol{w}^{\mu} - \eta\nabla_{\boldsymbol{w}}\mathcal{L}(\boldsymbol{w}^{\mu},\boldsymbol{v}^{\mu}|\boldsymbol{x}^{\mu},\boldsymbol{c}^{\mu}) = \boldsymbol{w}^{\mu} - \eta\left[\frac{\boldsymbol{x}^{\mu}\nabla_2\ell^{\mu}}{\sqrt{N}} + 2\frac{\boldsymbol{w}^{\mu}\nabla_3\ell^{\mu}}{N} + 2\frac{\boldsymbol{w}^{\mu}\nabla_1\tilde{g}^{\mu}}{N}\right] , \tag{S2}$$

$$\boldsymbol{v}^{\mu+1} = \boldsymbol{v}^{\mu} - \frac{\eta}{N}\nabla_4\ell^{\mu} - \frac{\eta}{N}\nabla_2\tilde{g}^{\mu} , \tag{S3}$$

where we use $\nabla_k\ell$ to denote the gradient of the function ℓ with respect to its kth argument, with the convention that it is reshaped as a matrix of the same dimensions of that argument, e.g., $\nabla_2\ell \in \mathbb{R}^{L\times K}$. For simplicity, we omit the function's arguments, by only keeping the time dependence, i.e., $\ell^{\mu} = \ell\left(\frac{\boldsymbol{x}^{\mu\top}\boldsymbol{w}_*}{\sqrt{N}},\frac{\boldsymbol{x}^{\mu\top}\boldsymbol{w}^{\mu}}{\sqrt{N}},\frac{\boldsymbol{w}^{\mu\top}\boldsymbol{w}^{\mu}}{N},\boldsymbol{v}^{\mu},\boldsymbol{c}^{\mu},z^{\mu}\right)$. For a given realization of the cluster coefficients $\boldsymbol{c}$, we introduce the compact notation $\boldsymbol{\mu}_{\boldsymbol{c}} \in \mathbb{R}^{N\times L}$ to denote the matrix with columns $\boldsymbol{\mu}_{l,c_l}$. It is useful to define the local fields

$$\boldsymbol{\lambda}^{\mu} = \frac{\boldsymbol{x}^{\mu\top}\boldsymbol{w}^{\mu}}{\sqrt{N}} \in \mathbb{R}^{L\times K} , \qquad \boldsymbol{\lambda}^{\mu}_* = \frac{\boldsymbol{x}^{\mu\top}\boldsymbol{w}_*}{\sqrt{N}} \in \mathbb{R}^{L\times M} , \qquad \boldsymbol{\rho}^{\mu}_{\boldsymbol{c}} = \frac{\boldsymbol{x}^{\mu\top}\boldsymbol{\mu}_{\boldsymbol{c}}}{\sqrt{N}} \in \mathbb{R}^{L\times L} . \tag{S4}$$

Notice that, due to the online-learning setup, at each training step the input $\boldsymbol{x}$ is independent of the weights. Therefore, due to the Gaussianity of the inputs, the local fields are also jointly Gaussian with zero mean and second moments given by:

$$\begin{aligned}\mathbb{E}_{\boldsymbol{x}|\boldsymbol{c}}\left[\lambda_{lk}\lambda_{l'k'}\right] &= \frac{\boldsymbol{w}_k\cdot\boldsymbol{\mu}_{l,c_l}}{N}\frac{\boldsymbol{w}_{k'}\cdot\boldsymbol{\mu}_{l',c_{l'}}}{N} + \delta_{l,l'}\,\sigma^2_{l,c_l}\frac{\boldsymbol{w}_k\cdot\boldsymbol{w}_{k'}}{N}\\ &= R_{k(l,c_l)}R_{k'(l',c_{l'})} + \delta_{l,l'}\sigma^2_{l,c_l}Q_{kk'} ,\end{aligned} \tag{S5}$$

$$\begin{aligned}\mathbb{E}_{\boldsymbol{x}|\boldsymbol{c}}\left[\lambda_{lk}\lambda_{*,l'm}\right] &= \frac{\boldsymbol{w}_k\cdot\boldsymbol{\mu}_{l,c_l}}{N}\frac{\boldsymbol{w}_{*,m}\cdot\boldsymbol{\mu}_{l',c_{l'}}}{N} + \delta_{l,l'}\,\sigma^2_{l,c_l}\frac{\boldsymbol{w}_k\cdot\boldsymbol{w}_{*,m}}{N}\\ &= R_{k(l,c_l)}S_{m(l',c_{l'})} + \delta_{l,l'}\sigma^2_{l,c_l}M_{km} ,\end{aligned} \tag{S6}$$

$$\begin{aligned}\mathbb{E}_{\boldsymbol{x}|\boldsymbol{c}}\left[\lambda_{*,lm}\lambda_{*,l'm'}\right] &= \frac{\boldsymbol{w}_{*,m}\cdot\boldsymbol{\mu}_{l,c_l}}{N}\frac{\boldsymbol{w}_{*,m'}\cdot\boldsymbol{\mu}_{l',c_{l'}}}{N} + \delta_{l,l'}\,\sigma^2_{l,c_l}\frac{\boldsymbol{w}_{*,m}\cdot\boldsymbol{w}_{*,m'}}{N}\\ &= S_{m(l,c_l)}S_{m'(l',c_{l'})} + \delta_{l,l'}\sigma^2_{l,c_l}T_{mm'} ,\end{aligned} \tag{S7}$$

$$
\begin{aligned}
\mathbb{E}_{\boldsymbol{x}|\boldsymbol{c}}\left[\lambda_{lk}\rho_{\boldsymbol{c}',l'l''}\right] &= \frac{\boldsymbol{w}_k\cdot\boldsymbol{\mu}_{l,c_l}}{N}\frac{\boldsymbol{\mu}_{l',c_{l'}}\cdot\boldsymbol{\mu}_{l'',c'_{l''}}}{N}+\delta_{l,l'}\,\sigma^2_{l,c_l}\frac{\boldsymbol{w}_k\cdot\boldsymbol{\mu}_{l'',c'_{l''}}}{N}\\
&= R_{k(l,c_l)}\Omega_{(l',c_{l'})(l'',c'_{l''})}+\delta_{l,l'}\sigma^2_{l,c_l}R_{k(l'',c'_{l''})}\ ,
\end{aligned}
\tag{S8}
$$

$$
\begin{aligned}
\mathbb{E}_{\boldsymbol{x}|\boldsymbol{c}}\left[\lambda_{*,lm}\rho_{\boldsymbol{c}',l'l''}\right] &= \frac{\boldsymbol{w}_{*,m}\cdot\boldsymbol{\mu}_{l,c_l}}{N}\frac{\boldsymbol{\mu}_{l',c_{l'}}\cdot\boldsymbol{\mu}_{l'',c'_{l''}}}{N}+\delta_{l,l'}\,\sigma^2_{l,c_l}\frac{\boldsymbol{w}_{*,m}\cdot\boldsymbol{\mu}_{l'',c'_{l''}}}{N}\\
&= S_{m(l,c_l)}\Omega_{(l',c_{l'})(l'',c'_{l''})}+\delta_{l,l'}\sigma^2_{l,c_l}S_{m(l'',c'_{l''})}\ ,
\end{aligned}
\tag{S9}
$$

$$
\begin{aligned}
\mathbb{E}_{\boldsymbol{x}|\boldsymbol{c}}\left[\rho_{\boldsymbol{c}',ll'}\rho_{\boldsymbol{c}'',l''l'''}\right] &= \frac{\boldsymbol{\mu}_{l',c'_{l'}}\cdot\boldsymbol{\mu}_{l,c_l}}{N}\frac{\boldsymbol{\mu}_{l'',c_{l''}}\cdot\boldsymbol{\mu}_{l''',c''_{l'''}}}{N}+\delta_{l,l''}\,\sigma^2_{l,c_l}\frac{\boldsymbol{\mu}_{l',c'_{l'}}\cdot\boldsymbol{\mu}_{l''',c''_{l'''}}}{N}\\
&= \Omega_{(l,c_l)(l',c'_{l'})}\Omega_{(l'',c_{l''})(l''',c''_{l'''})}+\delta_{l,l''}\sigma^2_{l,c_l}\Omega_{(l',c'_{l'})(l''',c''_{l'''})}\ ,
\end{aligned}
\tag{S10}
$$

where we have introduced the order parameters

$$
\begin{aligned}
&Q_{kk'}:=\frac{\boldsymbol{w}_k\cdot\boldsymbol{w}_{k'}}{N}\ ,\quad M_{km}:=\frac{\boldsymbol{w}^{\mu}_k\cdot\boldsymbol{w}_{*,m}}{N}\ ,\quad R_{k(l,c_l)}:=\frac{\boldsymbol{w}_k\cdot\boldsymbol{\mu}_{l,c_l}}{N}\ ,\\
&S_{m(l,c_l)}:=\frac{\boldsymbol{w}_{*,m}\cdot\boldsymbol{\mu}_{l,c_l}}{N}\ ,\quad T_{mm'}:=\frac{\boldsymbol{w}_{*,m}\cdot\boldsymbol{w}_{*,m'}}{N}\ ,\quad \Omega_{(l,c_l)(l',c'_{l'})}=\frac{\boldsymbol{\mu}_{l,c_l}\cdot\boldsymbol{\mu}_{l',c'_{l'}}}{N}\ .
\end{aligned}
\tag{S11}
$$

Note that in the expressions above the variable $\boldsymbol{x}$ is drawn with cluster membership $\boldsymbol{c}$ fixed. The additional cluster membership variables, e.g., $\boldsymbol{c}'$ and $\boldsymbol{c}''$ are fixed and do not intervene in the generative process of $\boldsymbol{x}$. The cost function defined in (S1) depends on the weights $\boldsymbol{w}$ only through the local fields and the order parameters. Similarly, the generalization error can be computed as an average over the local fields

$$
\varepsilon_g(\boldsymbol{w},\boldsymbol{v})=\mathbb{E}_{\boldsymbol{c}}\mathbb{E}_{(\boldsymbol{\lambda},\boldsymbol{\lambda}_*)|\boldsymbol{c}}\left[\ell_g\left(\boldsymbol{\lambda}_*,\boldsymbol{\lambda},\boldsymbol{Q},\boldsymbol{v},\boldsymbol{c},0\right)\right]\ ,
\tag{S12}
$$

where the function ℓ_g may coincide with the loss ℓ or denote a different metric depending on the context.

Since the local fields are Gaussian, their distribution is completely specified by the first two moments, which are functions of the order parameters. By substituting the update rules of (S2) into the definitions in (S11), we obtain the following evolution equations governing the order-parameter dynamics

$$
\begin{aligned}
\boldsymbol{Q}^{\mu+1}-\boldsymbol{Q}^{\mu} &= \frac{{\boldsymbol{w}^{\mu+1}}^{\top}\boldsymbol{w}^{\mu+1}}{N}-\frac{{\boldsymbol{w}^{\mu}}^{\top}\boldsymbol{w}^{\mu}}{N}=\\
&-\frac{\eta}{N}\left[{\boldsymbol{\lambda}^{\mu}}^{\top}\nabla_2\ell^{\mu}+\nabla_2{\ell^{\mu}}^{\top}\boldsymbol{\lambda}^{\mu}+2\boldsymbol{Q}^{\mu}\left(\nabla_3\ell^{\mu}+\nabla_1\tilde{g}^{\mu}\right)+2\left(\nabla_3\ell^{\mu}+\nabla_1\tilde{g}^{\mu}\right)^{\top}\boldsymbol{Q}^{\mu}\right]\\
&+\frac{\eta^2}{N}\left[\nabla_2{\ell^{\mu}}^{\top}\frac{{\boldsymbol{x}^{\mu}}^{\top}\boldsymbol{x}^{\mu}}{N}\nabla_2\ell^{\mu}+\mathcal{O}\left(\frac{1}{N}\right)\right]\ ,
\end{aligned}
\tag{S13}
$$

$$
\boldsymbol{M}^{\mu+1}-\boldsymbol{M}^{\mu}=\frac{{\boldsymbol{w}^{\mu+1}}^{\top}\boldsymbol{w}_*}{N}-\frac{{\boldsymbol{w}^{\mu}}^{\top}\boldsymbol{w}_*}{N}=-\frac{\eta}{N}\left[\nabla_2{\ell^{\mu}}^{\top}\boldsymbol{\lambda}^{\mu}_*+2\left(\nabla_3\ell^{\mu}+\nabla_1\tilde{g}^{\mu}\right)^{\top}\boldsymbol{M}^{\mu}\right]\ ,
\tag{S14}
$$

$$
\boldsymbol{R}^{\mu+1}_{\boldsymbol{c}'}-\boldsymbol{R}^{\mu}_{\boldsymbol{c}'}=\frac{{\boldsymbol{w}^{\mu+1}}^{\top}\boldsymbol{\mu}_{\boldsymbol{c}'}}{N}-\frac{{\boldsymbol{w}^{\mu}}^{\top}\boldsymbol{\mu}_{\boldsymbol{c}'}}{N}=-\frac{\eta}{N}\left[\nabla_2{\ell^{\mu}}^{\top}\boldsymbol{\rho}_{\boldsymbol{c}'}+2\left(\nabla_3\ell^{\mu}+\nabla_1\tilde{g}\right)^{\top}\boldsymbol{R}^{\mu}_{\boldsymbol{c}'}\right]\ ,
\tag{S15}
$$

where we have omitted subleading terms in N. Note that, while for convenience we write $\boldsymbol{R}_{\boldsymbol{c}'}$ for an arbitrary cluster membership variable $\boldsymbol{c}'=(c'_1,\ldots,c'_L)$, it is sufficient to keep track of the scalar variables $R_k,(l,c''_l)$ for $k=1,\ldots K$, $l=1,\ldots,L$, $c''_l=1,\ldots,C_l$, resulting in $K(C_1+C_2+\ldots+C_L)$ variables. We define a "training time" $\alpha=\mu/N$ and take the infinite-

dimensional limit $N \to \infty$ while keeping α of order one. We obtain the following ODEs

$$\begin{aligned}\frac{\mathrm{d}\boldsymbol{Q}}{\mathrm{d}\alpha} = \mathbb{E}_{\boldsymbol{c}}\Big[-\eta \Big\{ \mathbb{E}_{\boldsymbol{\lambda},\boldsymbol{\lambda}_*|\boldsymbol{c}} \Big[\boldsymbol{\lambda}^\top \nabla_2 \ell \Big] + 2\, \boldsymbol{Q} \left(\mathbb{E}_{\boldsymbol{\lambda},\boldsymbol{\lambda}_*|\boldsymbol{c}} [\nabla_3 \ell] + \nabla_1 \tilde{g} \right) + (\text{transpose}) \Big\} \\ + \eta^2\, \mathbb{E}_{\boldsymbol{\lambda},\boldsymbol{\lambda}_*|\boldsymbol{c}} \Big[\nabla_2 \ell^\top \mathrm{diag}(\boldsymbol{\sigma}^{\mathbf{2}}_{\boldsymbol{c}}) \nabla_2 \ell \Big] \Big] := f_{\boldsymbol{Q}}\ ,\end{aligned} \tag{S16}$$

$$\frac{\mathrm{d}\boldsymbol{M}}{\mathrm{d}\alpha} = \mathbb{E}_{\boldsymbol{c}}\Big[-\eta\, \mathbb{E}_{\boldsymbol{\lambda},\boldsymbol{\lambda}_*|\boldsymbol{c}} \Big[\nabla_2 \ell^\top \boldsymbol{\lambda}_* \Big] - 2\eta \left(\mathbb{E}_{\boldsymbol{\lambda},\boldsymbol{\lambda}_*|\boldsymbol{c}} [\nabla_3 \ell] + \nabla_1 \tilde{g} \right)^\top \boldsymbol{M} \Big] := f_{\boldsymbol{M}}\ , \tag{S17}$$

$$\frac{\mathrm{d}\boldsymbol{R}_{\boldsymbol{c}'}}{\mathrm{d}\alpha} = \mathbb{E}_{\boldsymbol{c}}\Big[-\eta\, \mathbb{E}_{\boldsymbol{\lambda},\boldsymbol{\lambda}_*|\boldsymbol{c}} \Big[\nabla_2 \ell^\top \boldsymbol{\rho}_{\boldsymbol{c}'} \Big] - 2\eta \left(\mathbb{E}_{\boldsymbol{\lambda},\boldsymbol{\lambda}_*|\boldsymbol{c}} [\nabla_3 \ell] + \nabla_1 \tilde{g} \right)^\top \boldsymbol{R}_{\boldsymbol{c}'} \Big] := f_{\boldsymbol{R}_{\boldsymbol{c}'}}\ , \tag{S18}$$

where we remind that $\ell = \ell\left(\boldsymbol{\lambda}_*, \boldsymbol{\lambda}, \boldsymbol{Q}, \boldsymbol{v}, \boldsymbol{c}, z\right)$ and $\tilde{g} = \tilde{g}(\boldsymbol{Q}, \boldsymbol{v})$, and we have defined the vector of variances $\boldsymbol{\sigma}^{\mathbf{2}}_{\boldsymbol{c}} = (\sigma^2_{1,c_1}, \ldots, \sigma^2_{L,c_L})$. In going from (S13) to (S16), we have used

$$\lim_{N\to\infty} \frac{\boldsymbol{x}_l \cdot \boldsymbol{x}_{l'}}{N} = \sigma^2_{l,c_l} \delta_{ll'}\ . \tag{S19}$$

Crucially, when taking the thermodynamic limit $N \to \infty$, we have replaced the right-hand sides in Eqs. (S16-S18) with their expected value over the data distribution. Indeed, it can be shown rigorously that, under additional assumptions, the fluctuations of the order parameters can be neglected [2]. Although we do not provide a rigorous proof of this result here, we verify this concentration property with numerical simulations, see Section S5. Finally, the additional parameters $\boldsymbol{v}$ evolve according to the low-dimensional equations

$$\frac{\mathrm{d}\boldsymbol{v}}{\mathrm{d}\alpha} = \mathbb{E}_{\boldsymbol{c}}\Big[-\eta\, \mathbb{E}_{\boldsymbol{\lambda},\boldsymbol{\lambda}_*|\boldsymbol{c}} [\nabla_4 \ell + \nabla_2 \tilde{g}] \Big] := f_{\boldsymbol{v}}\ . \tag{S20}$$

To conclude, note that the expectations in Eqs. (S16), (S18) and (S20) decompose into an average over the low-dimensional cluster vector $\mathbf{c}$, whose distribution is given by the model, and an average over the Gaussian fields $\boldsymbol{\lambda}$ and $\boldsymbol{\lambda}_*$, whose moments are fully specified by the order parameters, resulting in a closed-form system of equations. The expectations can be evaluated either analytically or via Monte Carlo sampling.

S1.1 Curriculum learning

The equations for the curriculum learning problem can be derived as a special case of those of [7]. The misclassification error can be expressed in terms of the order parameters as

$$\epsilon_g(\mathbb{Q}) = \frac{1}{2} - \frac{1}{\pi} \sin^{-1}\left(\frac{M_{11}}{\sqrt{T(Q_{11} + \Delta Q_{22})}} \right)\ . \tag{S21}$$

The evolution equations for the order parameters can be obtained from (S16) and (S18), yielding

$$\begin{aligned}
\frac{\mathrm{d}Q_{11}}{\mathrm{d}\alpha} &= -\bar{\lambda}Q_{11} + \frac{4\eta}{\pi(Q_{11}+\Delta Q_{22}+2)}\left[\frac{M_{11}(\Delta Q_{22}+2)}{\sqrt{T(Q_{11}+\Delta Q_{22}+2)-M_{11}^2}} - \frac{Q_{11}}{\sqrt{Q_{11}+\Delta Q_{22}+1}}\right] \\
&\quad + \frac{2}{\pi^2}\frac{\eta^2}{\sqrt{Q_{11}+\Delta Q_{22}+1}}\left[\frac{\pi}{2} + \sin^{-1}\left(\frac{Q_{11}+\Delta Q_{22}}{2+3(Q_{11}+\Delta Q_{22})}\right)\right. \\
&\quad \left. -2\sin^{-1}\left(\frac{M_{11}}{\sqrt{(3(Q_{11}+\Delta Q_{22})+2)}\sqrt{T(Q_{11}+\Delta Q_{22}+1)-M_{11}^2}}\right)\right], \\
\frac{\mathrm{d}Q_{22}}{\mathrm{d}\alpha} &= -\bar{\lambda}Q_{22} - \frac{4\eta\Delta Q_{22}}{\pi(Q_{11}+\Delta Q_{22}+2)}\left[\frac{M_{11}}{\sqrt{T(Q_{11}+\Delta Q_{22}+2)-M_{11}^2}} + \frac{1}{\sqrt{Q_{11}+\Delta Q_{22}+1}}\right] \\
&\quad + \frac{2}{\pi^2}\frac{\Delta\eta^2}{\sqrt{Q_{11}+\Delta Q_{22}+1}}\left[\frac{\pi}{2} + \sin^{-1}\left(\frac{Q_{11}+\Delta Q_{22}}{2+3(Q_{11}+\Delta Q_{22})}\right)\right. \\
&\quad \left. -2\sin^{-1}\left(\frac{M_{11}}{\sqrt{(3(Q_{11}+\Delta Q_{22})+2)}\sqrt{T(Q_{11}+\Delta Q_{22}+1)-M_{11}^2}}\right)\right], \\
\frac{\mathrm{d}M_{11}}{\mathrm{d}\alpha} &= -\frac{\bar{\lambda}}{2}M_{11} + \frac{2\eta}{\pi(Q_{11}+\Delta Q_{22}+2)}\left[\sqrt{T(Q_{11}+\Delta Q_{22}+2)-M_{11}^2} - \frac{M_{11}}{\sqrt{Q_{11}+\Delta Q_{22}+1}}\right],
\end{aligned} \tag{S22}$$

where $\bar{\lambda} = \lambda\eta$.

S1.2 Dropout regularization

In this section, we provide the expressions of the ODEs and the generalization error for the model of dropout regularization presented in the main text. This model corresponds to $L = C_1 = 1$, $\boldsymbol{\mu}_{1,1} = \mathbf{0}$, and $\sigma_{1,1} = 1$. The derivation of these results can be found in [5]. The generalization error reads

$$\begin{aligned}
\epsilon_g = \mathbb{E}_{\boldsymbol{x}}\left[\frac{1}{2}\left(f^*_{\boldsymbol{w}_*}(\boldsymbol{x}) - f^{\text{test}}_{\boldsymbol{w}}(\boldsymbol{x})\right)^2\right] &= \frac{p_f^2}{\pi}\sum_{i,k=1}^{K}\arcsin\left(\frac{Q_{ik}}{\sqrt{1+Q_{ii}}\sqrt{1+Q_{kk}}}\right) \\
+ \frac{1}{\pi}\sum_{n,m=1}^{K}\arcsin\left(\frac{T_{nm}}{\sqrt{1+T_{nn}}\sqrt{1+T_{mm}}}\right) &- \frac{2p_f}{\pi}\sum_{i=1}^{K}\sum_{n=1}^{M}\arcsin\left(\frac{M_{in}}{\sqrt{1+Q_{ii}}\sqrt{1+T_{nn}}}\right).
\end{aligned} \tag{S23}$$

The ODEs read

$$\frac{\mathrm{d}M_{in}}{\mathrm{d}\alpha} = f_{M_{in}}(Q,M), \qquad \frac{\mathrm{d}Q_{ik}}{\mathrm{d}\alpha} = f_{Q_{ik}}(Q,M), \tag{S24}$$

Introducing the notation

$$\mathcal{N}\left[p,\{i,j,k,\ldots,l\}\right] = p^n\,, \tag{S25}$$

where $n = |\{i, j, k, \ldots, l\}|$ is the cardinality of the set $\{i, j, k, \ldots, l\}$, we find [4]

$$f_{M_{in}} \equiv \eta \left[\sum_{m=1}^{M} \mathcal{N}\left[p, \{i\}\right] I_3(i, n, m) - \sum_{j=1}^{K} \mathcal{N}\left[p, \{i, j\}\right] I_3(i, n, j) \right], \tag{S26}$$

$$\begin{aligned} f_{Q_{ik}} \equiv\ & \eta \left[\sum_{m=1}^{M} \mathcal{N}\left[p, \{i\}\right] I_3(i, k, m) - \sum_{j=1}^{K} \mathcal{N}\left[p, \{i, j\}\right] I_3(i, k, j) \right] \\ & + \eta \left[\sum_{m=1}^{M} \mathcal{N}\left[p, \{k\}\right] I_3(k, i, m) - \sum_{j=1}^{K} \mathcal{N}\left[p, \{k, j\}\right] I_3(k, i, j) \right] \\ & + \eta^2 \left[\sum_{n=1}^{M} \sum_{m=1}^{M} \mathcal{N}\left[p, \{i, k\}\right] I_4(i, k, n, m) - 2 \sum_{j=1}^{K} \sum_{n=1}^{M} \mathcal{N}\left[p, \{i, k, j\}\right] I_4(i, k, j, n) \right. \\ & \left. + \sum_{j=1}^{K} \sum_{l=1}^{K} \mathcal{N}\left[p, \{i, j, k, l\}\right] I_4(i, k, j, l) + \mathcal{N}\left[p, \{i, k\}\right] \sigma^2 J_2(i, k) \right], \end{aligned} \tag{S27}$$

where

$$J_2 \equiv \frac{2}{\pi} \left(1 + c_{11} + c_{22} + c_{11}c_{22} - c_{12}^2\right)^{-1/2}, \tag{S28}$$

$$I_2 \equiv \frac{1}{\pi} \arcsin\left(\frac{c_{12}}{\sqrt{1 + c_{11}}\sqrt{1 + c_{12}}} \right), \tag{S29}$$

$$I_3 \equiv \frac{2}{\pi} \frac{1}{\sqrt{\Lambda_3}} \frac{c_{23}(1 + c_{11}) - c_{12}c_{13}}{1 + c_{11}}, \tag{S30}$$

$$I_4 \equiv \frac{4}{\pi^2} \frac{1}{\sqrt{\Lambda_4}} \arcsin\left(\frac{\Lambda_0}{\sqrt{\Lambda_1 \Lambda_2}} \right), \tag{S31}$$

and

$$\Lambda_4 = (1 + c_{11})(1 + c_{22}) - c_{12}^2, \tag{S32}$$

$$\Lambda_3 = (1 + c_{11}) * (1 + c_{33}) - c_{13}^2, \tag{S33}$$

$$\Lambda_0 = \Lambda_4 c_{34} - c_{23}c_{24}(1 + c_{11}) - c_{13}c_{14}(1 + c_{22}) + c_{12}c_{13}c_{24} + c_{12}c_{14}c_{23}, \tag{S34}$$

$$\Lambda_1 = \Lambda_4(1 + c_{33}) - c_{23}^2(1 + c_{11}) - c_{13}^2(1 + c_{22}) + 2c_{12}c_{13}c_{23}, \tag{S35}$$

$$\Lambda_2 = \Lambda_4(1 + c_{44}) - c_{24}^2(1 + c_{11}) - c_{14}^2(1 + c_{22}) + 2c_{12}c_{14}c_{24}. \tag{S36}$$

The indices i, j, k, l and n, m indicate the student's and the teacher's nodes, respectively. For compactness, we adopt the notation for I_2, I_3, and I_4 of Ref. [2]. As an example, $I(i, n)$ takes as input the correlation matrix of the preactivations corresponding to the indices i and n, i.e., $\lambda_i = \boldsymbol{w}_i \cdot \boldsymbol{x}/\sqrt{N}$ and $\lambda_{*,n} = \boldsymbol{w}_n^* \cdot \boldsymbol{x}/\sqrt{N}$. For this example, the correlation matrix would be

$$C = \begin{pmatrix} c_{11} & c_{12} \\ c_{21} & c_{22} \end{pmatrix} = \begin{pmatrix} \langle \lambda_i \lambda_i \rangle & \langle \lambda_i \lambda_{*,n} \rangle \\ \langle \lambda_{*,n} \lambda_i \rangle & \langle \lambda_{*,n} \lambda_{*,n} \rangle \end{pmatrix} = \begin{pmatrix} Q_{ii} & M_{in} \\ M_{in} & T_{nn} \end{pmatrix}. \tag{S37}$$

S1.3 Denoising autoencoder

We define the additional local fields

$$\tilde{\lambda}_k \equiv \frac{\tilde{\boldsymbol{x}} \cdot \boldsymbol{w}_k}{\sqrt{N}} = \sqrt{1 - \Delta}\lambda_{1,k} + \sqrt{\Delta}\lambda_{2,k}, \quad \tilde{\rho}_{\boldsymbol{c},l} \equiv \frac{\tilde{\boldsymbol{x}} \cdot \boldsymbol{\mu}_{l,c_l}}{\sqrt{N}} = \sqrt{1 - \Delta}\rho_{\boldsymbol{c},1l} + \sqrt{\Delta}\rho_{\boldsymbol{c},2l}, \tag{S38}$$

where we recall $\lambda_{1,k} = \boldsymbol{w}_k \cdot \boldsymbol{x}_1/\sqrt{N}$, $\lambda_{2,k} = \boldsymbol{w}_k \cdot \boldsymbol{x}_2/\sqrt{N}$, $\rho_{\boldsymbol{c},1l} = \boldsymbol{\mu}_{l,c_l} \cdot \boldsymbol{x}_1/\sqrt{N}$, $\rho_{\boldsymbol{c},2l} = \boldsymbol{\mu}_{l,c_l} \cdot \boldsymbol{x}_2/\sqrt{N}$. Here, we take $C_2 = 1$ and $\boldsymbol{\mu}_{2,c_2} = \boldsymbol{0}$, so that $\rho_{\boldsymbol{c},12} = \rho_{\boldsymbol{c},22} = \tilde{\rho}_{\boldsymbol{c},2} = 0$. The local fields are Gaussian variables with moments given by

$$\mathbb{E}_{\boldsymbol{x}|\boldsymbol{c}}\left[\lambda_{1,k}\right] = \frac{\boldsymbol{w}_k \cdot \boldsymbol{\mu}_{1,c_1}}{N} = R_{k(1,c_1)}\,, \qquad \mathbb{E}_{\boldsymbol{x}|\boldsymbol{c}}\left[\rho_{\boldsymbol{c}',11}\right] = \frac{\boldsymbol{\mu}_{1,c_1} \cdot \boldsymbol{\mu}_{1,c_1'}}{N} = \Omega_{(1,c_1)(1,c_1')}\,, \tag{S39}$$

$$\mathbb{E}_{\boldsymbol{x}|\boldsymbol{c}}\left[\lambda_{2,k}\right] = \mathbb{E}_{\boldsymbol{x}|\boldsymbol{c}}\left[\rho_{\boldsymbol{c}',2l}\right] = 0\,, \tag{S40}$$

$$\mathbb{E}_{\boldsymbol{x}|\boldsymbol{c}}\left[\lambda_{1,k}\lambda_{2,h}\right] = \mathbb{E}_{\boldsymbol{x}|\boldsymbol{c}}\left[\lambda_{1,k}\rho_{\boldsymbol{c}',2l}\right] = \mathbb{E}_{\boldsymbol{x}|\boldsymbol{c}}\left[\lambda_{2,k}\rho_{\boldsymbol{c}',1l}\right] = \mathbb{E}_{\boldsymbol{x}|\boldsymbol{c}}\left[\rho_{\boldsymbol{c}',1l}\rho_{\boldsymbol{c}',2l'}\right] = 0\,, \tag{S41}$$

$$\mathbb{E}_{\boldsymbol{x}|\boldsymbol{c}}\left[\lambda_{1,k}\lambda_{1,h}\right] = R_{k(1,c_1)}R_{h(1,c1)} + \sigma^2_{1,c_1}Q_{kh}\,, \qquad \mathbb{E}_{\boldsymbol{x}|\boldsymbol{c}}\left[\lambda_{2,k}\lambda_{2,h}\right] = Q_{kh}\,, \tag{S42}$$

$$\mathbb{E}_{\boldsymbol{x}|\boldsymbol{c}}\left[\tilde{\lambda}_j\lambda_{1,k}\right] = \sqrt{1-\Delta}\,\mathbb{E}_{\boldsymbol{x}|\boldsymbol{c}}\left[\lambda_{1,k}\lambda_{1,j}\right]\,, \qquad \mathbb{E}_{\boldsymbol{x}|\boldsymbol{c}}\left[\tilde{\lambda}_j\lambda_{2,k}\right] = \sqrt{\Delta}\,\mathbb{E}_{\boldsymbol{x}|\boldsymbol{c}}\left[\lambda_{2,k}\lambda_{2,j}\right]\,, \tag{S43}$$

$$\mathbb{E}_{\boldsymbol{x}|\boldsymbol{c}}\left[\rho^2_{\boldsymbol{c}',11}\right] = \Omega^2_{(1,c_1)(1,c_1')} + \sigma^2_{1,c_1}\Omega_{(1,c_1')(1,c_1)}\,, \qquad \mathbb{E}_{\boldsymbol{x}|\boldsymbol{c}}\left[\rho^2_{\boldsymbol{c}',21}\right] = \Omega_{(1,c_1')(1,c_1')}\,. \tag{S44}$$

$$\mathbb{E}_{\boldsymbol{x}|\boldsymbol{c}}\left[\lambda_{1,k}\rho_{\boldsymbol{c}',11}\right] = \sigma^2_{1,c_1}R_{k(1,c_1')} + \Omega_{(1,c_1')(1,c_1)}R_{k(1,c_1)}\,, \qquad \mathbb{E}_{\boldsymbol{x}|\boldsymbol{c}}\left[\lambda_{2,k}\rho_{\boldsymbol{c}',21}\right] = R_{k(1,c_1')}\,. \tag{S45}$$

It is also useful to compute the first moments of the combined variables

$$\mathbb{E}_{\boldsymbol{x}|\boldsymbol{c}}\left[\tilde{\lambda}_k\right] = \sqrt{1-\Delta}\,R_{k(1,c_1)}\,, \qquad \mathbb{E}_{\boldsymbol{x}|\boldsymbol{c}}\left[\tilde{\rho}_{\boldsymbol{c}',1}\right] = \sqrt{1-\Delta}\,\Omega_{(1,c_1)(1,c_1')}\,, \tag{S46}$$

and the second moments

$$\begin{aligned}\mathbb{E}_{\boldsymbol{x}|\boldsymbol{c}}\left[\tilde{\lambda}_k\tilde{\lambda}_h\right] - \mathbb{E}_{\boldsymbol{x}|\boldsymbol{c}}\left[\tilde{\lambda}_k\right]\mathbb{E}_{\boldsymbol{x}|\boldsymbol{c}}\left[\tilde{\lambda}_h\right] &= \left[(1-\Delta)\sigma^2_{1,c_1} + \Delta\right]Q_{kh}\,,\\ \mathbb{E}_{\boldsymbol{x}|\boldsymbol{c}}\left[\tilde{\rho}^2_{\boldsymbol{c}',1}\right] - \mathbb{E}_{\boldsymbol{x}|\boldsymbol{c}}\left[\tilde{\rho}_{\boldsymbol{c}',1}\right]^2 &= \left[(1-\Delta)\sigma^2_{1,c_1} + \Delta\right]\Omega_{(1,c_1')(1,c_1')}\,.\end{aligned} \tag{S47}$$

Finally, we have

$$\mathbb{E}_{\boldsymbol{x}|\boldsymbol{c}}\left[\tilde{\lambda}_k\rho_{\boldsymbol{c}',11}\right] = \sqrt{1-\Delta}\,\mathbb{E}_{\boldsymbol{x}|\boldsymbol{c}}\left[\lambda_{1,k}\rho_{\boldsymbol{c}',11}\right]\,, \tag{S48}$$

$$\mathbb{E}_{\boldsymbol{x}|\boldsymbol{c}}\left[\tilde{\lambda}_k\tilde{\rho}_{\boldsymbol{c}',1}\right] = (1-\Delta)\mathbb{E}_{\boldsymbol{x}|\boldsymbol{c}}\left[\lambda_{1,k}\rho_{\boldsymbol{c}',11}\right] + \Delta\mathbb{E}_{\boldsymbol{x}|\boldsymbol{c}}\left[\lambda_{2,k}\rho_{\boldsymbol{c}',21}\right]\,. \tag{S49}$$

The mean squared error (MSE) can be expressed in terms of the order parameter as follows

$$\begin{aligned}\mathrm{MSE}(\boldsymbol{w},b) = \mathbb{E}_{\boldsymbol{x},\boldsymbol{c}}\left[\|\boldsymbol{x} - f_{\boldsymbol{w},b}(\tilde{\boldsymbol{x}})\|_2^2\right] &= \mathbb{E}_{\boldsymbol{c}}\left\{N\left[\sigma_k^2\left(1 - b\sqrt{1-\Delta}\right)^2 + b^2\Delta\right]\right.\\ &\left.+ \sum_{j,k=1}^{K} Q_{jk}\mathbb{E}_{\boldsymbol{x}|\boldsymbol{c}}\left[g(\tilde{\lambda}_j)g(\tilde{\lambda}_k)\right] - 2\sum_{k=1}^{K}\mathbb{E}_{\boldsymbol{x}|\boldsymbol{c}}\left[(\lambda_{1k} - b\tilde{\lambda}_k)g(\tilde{\lambda}_k)\right]\right\}\,,\end{aligned} \tag{S50}$$

where we have neglected constant terms. The weights are updated according to

$$\begin{aligned}\boldsymbol{w}_k^{\mu+1} = \boldsymbol{w}_k^{\mu} &+ \frac{\eta}{\sqrt{N}}g\left(\tilde{\lambda}_k^{\mu}\right)\left(\boldsymbol{x}_1^{\mu} - b\,\tilde{\boldsymbol{x}}^{\mu} - \sum_{h=1}^{K}\frac{\boldsymbol{w}_h^{\mu}}{\sqrt{N}}g\left(\tilde{\lambda}_h^{\mu}\right)\right)\\ &+ \frac{\eta}{\sqrt{N}}g'(\tilde{\lambda}_k^{\mu})\left(\lambda_{1,k}^{\mu} - b\,\tilde{\lambda}_k^{\mu} - \sum_{h=1}^{K}\frac{\boldsymbol{w}_k^{\mu}\cdot\boldsymbol{w}_h^{\mu}}{N}g\left(\tilde{\lambda}_h^{\mu}\right)\right)\tilde{\boldsymbol{x}}^{\mu}\,,\end{aligned} \tag{S51}$$

The skip connection is also trained with SGD. To leading order, we find

$$b^{\mu+1} = b^\mu + \frac{\eta_b}{N}\left(\sqrt{1-\Delta}\sigma_{1,c_1}^2 - b^\mu(1-\Delta)\sigma_{1,c_1}^2 - b^\mu\Delta\right) . \tag{S52}$$

Note that, conditioning on a given cluster c_1, for large N, we have

$$\frac{1}{N}\boldsymbol{x}_1\cdot\boldsymbol{x}_1 \underset{N\gg1}{\approx} \sigma_{1,c_1}^2\,, \quad \frac{1}{N}\tilde{\boldsymbol{x}}\cdot\tilde{\boldsymbol{x}} \underset{N\gg1}{\approx} (1-\Delta)\sigma_{1,c_1}^2+\Delta\,, \quad \frac{1}{N}\boldsymbol{x}_1\cdot\tilde{\boldsymbol{x}} \underset{N\gg1}{\approx} \sqrt{1-\Delta}\,\sigma_{1,c_1}^2\,. \tag{S53}$$

For simplicity, we will consider the linear activation $g(z) = z$. In this case, it is possible to derive explicit equations for the evolution of the order parameters as follows:

$$\begin{aligned} R_{k(1,c_1')}^{\mu+1} = R_{k(1,c_1')}^{\mu} + \frac{\eta}{N}\mathbb{E}_{\boldsymbol{c}}\Bigg[&\mathbb{E}_{\boldsymbol{x}|\boldsymbol{c}}\left[\tilde\lambda_k^\mu\rho_{\boldsymbol{c'},11}^\mu\right] - 2b\mathbb{E}_{\boldsymbol{x}|\boldsymbol{c}}\left[\tilde\lambda_k^\mu\tilde\rho_{\boldsymbol{c'},1}^\mu\right] - \sum_{j=1}^K R_{j(1,c_1')}^\mu \mathbb{E}_{\boldsymbol{x}|\boldsymbol{c}}\left[\tilde\lambda_k^\mu\tilde\lambda_j^\mu\right] \\ &+ \mathbb{E}_{\boldsymbol{x}|\boldsymbol{c}}\left[\lambda_{1,k}^\mu\tilde\rho_{\boldsymbol{c'},1}^\mu\right] - \sum_{j=1}^K Q_{jk}\mathbb{E}_{\boldsymbol{x}|\boldsymbol{c}}\left[\tilde\lambda_j^\mu\tilde\rho_{\boldsymbol{c'},1}\right]\Bigg] \,, \end{aligned} \tag{S54}$$

$$\begin{aligned} Q_{jk}^{\mu+1} &= Q_{jk}^\mu + \frac{\eta}{N}\mathbb{E}_{\boldsymbol{c}}\Bigg\{\left(\mathbb{E}_{\boldsymbol{x}|\boldsymbol{c}}\left[\tilde\lambda_j\Lambda_k\right] + \mathbb{E}_{\boldsymbol{x}|\boldsymbol{c}}\left[\tilde\lambda_k\Lambda_j\right]\right)\left[2+\eta\left(\frac{\boldsymbol{x}_1\cdot\tilde{\boldsymbol{x}}}{N} - b\frac{\tilde{\boldsymbol{x}}\cdot\tilde{\boldsymbol{x}}}{N}\right)\right] + \eta\mathbb{E}_{\boldsymbol{x}|\boldsymbol{c}}\left[\Lambda_j\Lambda_k\right]\frac{\tilde{\boldsymbol{x}}\cdot\tilde{\boldsymbol{x}}}{N} \\ &\quad + \eta\mathbb{E}_{\boldsymbol{x}|\boldsymbol{c}}\left[\tilde\lambda_j\tilde\lambda_k\right]\left(\frac{\boldsymbol{x}_1\cdot\boldsymbol{x}_1}{N} - 2b\frac{\boldsymbol{x}_1\cdot\tilde{\boldsymbol{x}}}{N} + b^2\frac{\tilde{\boldsymbol{x}}\cdot\tilde{\boldsymbol{x}}}{N}\right)\Bigg\} \\ &= Q_{jk}^\mu + \frac{\eta}{N}\mathbb{E}_{\boldsymbol{c}}\Bigg\{\left(\mathbb{E}_{\boldsymbol{x}|\boldsymbol{c}}\left[\tilde\lambda_j\Lambda_k\right] + \mathbb{E}_{\boldsymbol{x}|\boldsymbol{c}}\left[\tilde\lambda_k\Lambda_j\right]\right)\left[2+\eta\left(\sqrt{1-\Delta}\sigma_{1,c_1}^2 - b((1-\Delta)\sigma_{1,c_1}^2+\Delta)\right)\right] \\ &\quad + \eta\mathbb{E}_{\boldsymbol{x}|\boldsymbol{c}}\left[\Lambda_j\Lambda_k\right]((1-\Delta)\sigma_{1,c_1}^2+\Delta) + \eta\mathbb{E}_{\boldsymbol{x}|\boldsymbol{c}}\left[\tilde\lambda_j\tilde\lambda_k\right]\left(\sigma_{1,c_1}^2 - 2b\sqrt{1-\Delta}\,\sigma_{1,c_1}^2 + b^2((1-\Delta)\sigma_{1,c_1}^2+\Delta)\right)\Bigg\} \end{aligned} \tag{S55}$$

where we have introduced the definition

$$\Lambda_k \equiv \lambda_{1,k} - b\tilde\lambda_k - \sum_{j=1}^K Q_{jk}\tilde\lambda_j\,. \tag{S56}$$

We can compute the averages

$$\begin{aligned} \mathbb{E}_{\boldsymbol{x}|\boldsymbol{c}}\left[\tilde\lambda_j\Lambda_k\right] &= \mathbb{E}_{\boldsymbol{x}|\boldsymbol{c}}\left[\tilde\lambda_j\lambda_{1,k}\right] - \sum_{i=1}^K\left(b\delta_{ik}+Q_{ki}\right)\mathbb{E}_{\boldsymbol{x}|\boldsymbol{c}}\left[\tilde\lambda_j\tilde\lambda_i\right]\,, \\ \mathbb{E}_{\boldsymbol{x}|\boldsymbol{c}}\left[\Lambda_j\Lambda_k\right] &= \mathbb{E}_{\boldsymbol{x}|\boldsymbol{c}}\left[\lambda_{1,j}\lambda_{1,k}\right] - \sum_{i=1}^K\left(b\delta_{ij}+Q_{ji}\right)\mathbb{E}_{\boldsymbol{x}|\boldsymbol{c}}\left[\tilde\lambda_i\lambda_{1,k}\right] - \sum_{i=1}^K\left(b\delta_{ik}+Q_{ki}\right)\mathbb{E}_{\boldsymbol{x}|\boldsymbol{c}}\left[\tilde\lambda_i\lambda_{1,j}\right] \\ &\quad + \sum_{i,\ell=1}^K\left(b\delta_{ik}+Q_{ki}\right)\left(b\delta_{\ell j}+Q_{j\ell}\right)\mathbb{E}_{\boldsymbol{x}|\boldsymbol{c}}\left[\tilde\lambda_i\tilde\lambda_\ell\right]\,. \end{aligned} \tag{S57}$$

Finally, it is useful to evaluate the MSE in the special case of linear activation:

$$\begin{aligned} \mathrm{MSE} = \mathbb{E}_{\boldsymbol{c}}\Bigg\{&N\left[\sigma_{1,c_1}^2\left(1-b\sqrt{1-\Delta}\right)^2 + b^2\Delta\right] \\ &+ \sum_{j,k=1}^K Q_{jk}\left[\left((1-\Delta)\sigma_{1,c_1}^2+\Delta\right)Q_{jk} + (1-\Delta)R_{j,(1,c_1)}R_{k,(1,c_1)}\right] \\ &- 2\sum_{k=1}^K\left[\sqrt{1-\Delta}\sigma_{1,c_1}^2 Q_{kk} - b\left[\left((1-\Delta)\sigma_{1,c_1}^2+\Delta\right)Q_{kk} + (1-\Delta)R_{k,(1,c_1)}^2\right]\right]\Bigg\}\,. \end{aligned} \tag{S58}$$

S1.3.1 Data augmentation

We consider inputs $\boldsymbol{x} = (\boldsymbol{x}_1, \boldsymbol{x}_2, \ldots, \boldsymbol{x}_{B+1}) \in \mathbb{R}^{N\times B+1}$, where $\boldsymbol{x}_1 \sim \mathcal{N}\left(\frac{\boldsymbol{\mu}_{1,c_1}}{\sqrt{N}}, \sigma^2 \boldsymbol{I}_N\right)$ denotes the clean input and $\boldsymbol{x}_2, \ldots, \boldsymbol{x}_{B+1} \overset{\text{i.i.d.}}{\sim} \mathcal{N}(\boldsymbol{0}, \boldsymbol{I}_N)$. Each clean input $\boldsymbol{x}_1$ is used to create multiple corrupted samples: $\tilde{\boldsymbol{x}}_a = \sqrt{1-\Delta}\,\boldsymbol{x}_1 + \sqrt{\Delta}\,\boldsymbol{x}_{a+1}$, $a = 1, \ldots, B$, that are used as a mini-batch for training. The SGD dynamics of the tied weights modifies as follows:

$$\boldsymbol{w}_k^{\mu+1} = \boldsymbol{w}_k^{\mu} + \frac{\eta}{B^\mu\sqrt{N}} \sum_{a=1}^{B^\mu} \left\{ \tilde{\lambda}_{a,k}^{\mu} \left(\boldsymbol{x}_1^\mu - b\,\tilde{\boldsymbol{x}}_a^\mu - \sum_{j=1}^{K} \frac{\boldsymbol{w}_j^\mu}{\sqrt{N}} \tilde{\lambda}_{a,j}^{\mu} \right) + \left(\lambda_{1,k}^\mu - b\,\tilde{\lambda}_{a,k}^\mu - \sum_{j=1}^{K} \frac{\boldsymbol{w}_k^\mu \cdot \boldsymbol{w}_j^\mu}{N} \tilde{\lambda}_{a,j}^\mu \right) \tilde{\boldsymbol{x}}_a^\mu \right\}, \tag{S59}$$

where

$$\tilde{\lambda}_{a,k} = \frac{\tilde{\boldsymbol{x}}_a \cdot \boldsymbol{w}_k}{\sqrt{N}} = \sqrt{1-\Delta}\lambda_{1,k} + \sqrt{\Delta}\lambda_{a+1,k}\,. \tag{S60}$$

While the equations for b and M remain unchanged, we need to include additional terms in the equation for Q. We find

$$\begin{aligned}
Q_{jk}^{\mu+1} = Q_{jk}^{\mu} &+ \frac{\eta}{N}\mathbb{E}_{\boldsymbol{c}} \Big\{ \left(\mathbb{E}_{\boldsymbol{x}|\boldsymbol{c}}\left[\tilde{\lambda}_j \Lambda_k\right] + \mathbb{E}_{\boldsymbol{x}|\boldsymbol{c}}\left[\tilde{\lambda}_k \Lambda_j\right] \right) \left[2 + \frac{\eta}{B} \left(\sqrt{1-\Delta}\sigma_{1,c_1}^2 - b((1-\Delta)\sigma_{1,c_1}^2 + \Delta) \right) \right] \\
&+ \frac{\eta}{B}\mathbb{E}_{\boldsymbol{x}|\boldsymbol{c}}\left[\Lambda_j\Lambda_k\right]((1-\Delta)\sigma_{1,c_1}^2 + \Delta) \\
&+ \frac{\eta}{B}\mathbb{E}_{\boldsymbol{x}|\boldsymbol{c}}\left[\tilde{\lambda}_j\tilde{\lambda}_k\right] \left(\sigma_{1,c_1}^2 - 2b\sqrt{1-\Delta}\,\sigma_{1,c_1}^2 + b^2((1-\Delta)\sigma_{1,c_1}^2 + \Delta) \right) \\
&+ \frac{\eta(B-1)}{B}(1-\Delta)\mathbb{E}_{\boldsymbol{x}|\boldsymbol{c}}\left[\Lambda_{a,j}\Lambda_{a',k}\right]\sigma_{1,c_1}^2 \\
&+ \frac{\eta(B-1)}{B}\mathbb{E}_{\boldsymbol{x}|\boldsymbol{c}}\left[\tilde{\lambda}_{a,j}\tilde{\lambda}_{a',k}\right] \left(\left(1 + b^2(1-\Delta)\right)\sigma_{1,c_1}^2 - 2b\sqrt{1-\Delta}\sigma_{1,c_1}^2 \right) \\
&+ \frac{\eta(B-1)}{B} \left(\mathbb{E}_{\boldsymbol{x}|\boldsymbol{c}}\left[\tilde{\lambda}_{a,j}\Lambda_{a',k}\right] + \mathbb{E}_{\boldsymbol{x}|\boldsymbol{c}}\left[\tilde{\lambda}_{a,k}\Lambda_{a',j}\right] \right) \left(\sqrt{1-\Delta}\sigma_{1,c_1}^2 - b(1-\Delta)\sigma_{1,c_1}^2 \right) \Big\}
\end{aligned} \tag{S61}$$

We derive the following expressions for the average quantities, valid for $a \neq a'$

$$\mathbb{E}_{\boldsymbol{x}|\boldsymbol{c}}\left[\tilde{\lambda}_{a,j}\tilde{\lambda}_{a',k}\right] = (1-\Delta)\mathbb{E}_{\boldsymbol{x}|\boldsymbol{c}}\left[\lambda_{1,j}\lambda_{1,k}\right], \tag{S62}$$

$$\mathbb{E}_{\boldsymbol{x}|\boldsymbol{c}}\left[\tilde{\lambda}_{a,j}\Lambda_{a',k}\right] = \left[\sqrt{1-\Delta} - b(1-\Delta)\right]\mathbb{E}_{\boldsymbol{x}|\boldsymbol{c}}\left[\lambda_{1,j}\lambda_{1,k}\right] - (1-\Delta)\sum_{i=1}^{K} Q_{ki}\mathbb{E}_{\boldsymbol{x}|\boldsymbol{c}}\left[\lambda_{1,j}\lambda_{1,i}\right], \tag{S63}$$

$$\begin{aligned}
\mathbb{E}_{\boldsymbol{x}|\boldsymbol{c}}\left[\Lambda_{a,j}\Lambda_{a',k}\right] = (1 - b\sqrt{1-\Delta})^2\mathbb{E}_{\boldsymbol{x}|\boldsymbol{c}}\left[\lambda_{1,j}\lambda_{1,k}\right] + (1-\Delta)\sum_{i,h=1}^{K} Q_{ji}Q_{kh}\mathbb{E}_{\boldsymbol{x}|\boldsymbol{c}}\left[\lambda_{1,i}\lambda_{1,h}\right] \\
+ \left[b(1-\Delta) - \sqrt{1-\Delta}\right]\sum_{i=1}^{K}\left(Q_{ji}\mathbb{E}_{\boldsymbol{x}|\boldsymbol{c}}\left[\lambda_{1,k}\lambda_{1,i}\right] + Q_{ki}\mathbb{E}_{\boldsymbol{x}|\boldsymbol{c}}\left[\lambda_{1,j}\lambda_{1,i}\right] \right),
\end{aligned} \tag{S64}$$

where $\Lambda_{a,j}$ is defined as in (S56).

S2 Optimal control methods

S2.1 Indirect methods

We consider the cost functional

$$\mathcal{F}[\boldsymbol{u}] = \epsilon_g(\mathbb{Q}(\alpha_F)), \tag{S65}$$

where the square brackets indicate functional dependence on the full control trajectory $\boldsymbol{u}(\alpha)$, for $0 \le \alpha \le \alpha_F$. Following Pontryagin's maximum principle [6], we introduce the Lagrange multipliers $\hat{\mathbb{Q}}(\alpha)$ to enforce the training dynamics

$$\frac{\mathrm{d}\mathbb{Q}(\alpha)}{\mathrm{d}\alpha} = f_{\mathbb{Q}}\big(\mathbb{Q}(\alpha),\, \boldsymbol{u}(\alpha)\big)\,. \tag{S66}$$

We obtain

$$\mathcal{F}[\boldsymbol{u}, \mathbb{Q}, \hat{\mathbb{Q}}] = \epsilon_g\big(\mathbb{Q}(\alpha_F)\big) + \int_0^{\alpha_F} \mathrm{d}\alpha\, \hat{\mathbb{Q}}(\alpha) \cdot \left[-\frac{\mathrm{d}\mathbb{Q}(\alpha)}{\mathrm{d}\alpha} + f_{\mathbb{Q}}\big(\mathbb{Q}(\alpha),\, \boldsymbol{u}(\alpha)\big)\right], \tag{S67}$$

where $\hat{\mathbb{Q}}(\alpha)$ are known as adjoint (or costate) variables. The optimality conditions are $\delta\mathcal{F}/\delta\hat{\mathbb{Q}}(\alpha) = 0$ and $\delta\mathcal{F}/\delta\mathbb{Q}(\alpha) = 0$. The first yields the forward dynamics (S66). For $\alpha < \alpha_F$, the second, after integration by parts, gives the adjoint (backward) ODEs

$$-\frac{\mathrm{d}\hat{\mathbb{Q}}(\alpha)^\top}{\mathrm{d}\alpha} = \hat{\mathbb{Q}}(\alpha)^\top \nabla_{\mathbb{Q}} f_{\mathbb{Q}}\big(\mathbb{Q}(\alpha), \boldsymbol{u}(\alpha)\big), \tag{S68}$$

with the final condition at $\alpha = \alpha_F$:

$$\hat{\mathbb{Q}}(\alpha_F) = \nabla_{\mathbb{Q}}\, \epsilon_g\big(\mathbb{Q}(\alpha_F)\big). \tag{S69}$$

Variations at $\alpha = 0$ are not considered since $\mathbb{Q}(0) = \mathbb{Q}_0$ is fixed. Finally, optimizing $\boldsymbol{u}$ point-wise yields

$$\boldsymbol{u}^*(\alpha) = \underset{\boldsymbol{u}\in\mathcal{U}}{\arg\min} \left\{\hat{\mathbb{Q}}(\alpha) \cdot f_{\mathbb{Q}}\big(\mathbb{Q}(\alpha), \boldsymbol{u}\big)\right\}. \tag{S70}$$

In practice, we use the forward-backward sweep method: starting from an initial guess for $\boldsymbol{u}$, we iterate the following steps until convergence.

1. Integrate $\mathbb{Q}$ forward via (S66) from $\mathbb{Q}(0) = \mathbb{Q}_0$.
2. Integrate $\hat{\mathbb{Q}}$ backward via (S68) from $\hat{\mathbb{Q}}(\alpha_F)$ in (S69).
3. Update $\boldsymbol{u}^{k+1}(\alpha) = \gamma_{\mathrm{damp}}\boldsymbol{u}^k(\alpha) + (1-\gamma_{\mathrm{damp}})\boldsymbol{u}^*(\alpha)$, where $\boldsymbol{u}^*(\alpha)$ is given in (S70).

We typically choose the damping parameter $\gamma_{\mathrm{damp}} > 0.9$. Convergence is usually reached within a few hundred to a few thousand iterations.

S2.2 Direct methods

Direct methods discretize the control trajectory $\boldsymbol{u}(\alpha)$ on a finite grid of $I = \alpha_F/\mathrm{d}\alpha$ intervals and map the continuous-time OC problem into a finite-dimensional nonlinear program (NLP). We introduce optimization variables for $\mathbb{Q}$ and $\boldsymbol{u}$ at each node $\alpha_j = j\,\mathrm{d}\alpha$, enforce the dynamics (S66) via constraints on each interval, and solve the resulting NLP using the CasADi package [1]. In this paper, we implement a multiple-shooting scheme: $\boldsymbol{u}(\alpha)$ is parameterized as constant on each interval, and continuity of $\mathbb{Q}$ is enforced at the boundaries. While direct methods are conceptually simpler—relying on standard NLP solvers and avoiding the explicit derivation of adjoint equations—in the settings under consideration, we find that they tend to perform worse when the control $\boldsymbol{u}$ has discrete components. Conversely, indirect methods require computing costate derivatives but yield more accurate solutions for discrete controls. Depending on the problem setting, we therefore choose between direct and indirect approaches as specified in each case. Numerical implementations of both methods are available at [3].

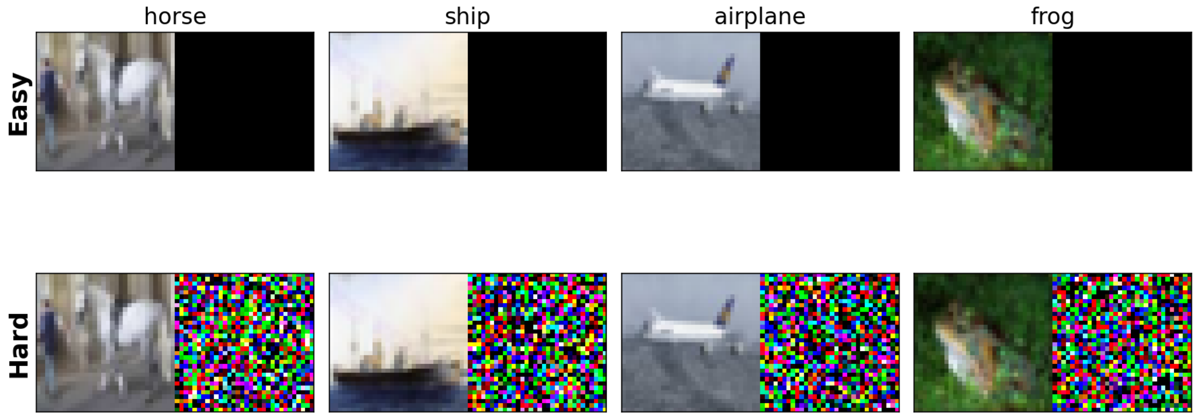

Figure S1: Example composite images from the Cluttered CIFAR-10 dataset. The left half of each composite is the target, while the right half is the distractor. **Top row (Easy)**: the distractor is black ($\sigma = 0$). **Bottom row (Hard)**: the distractor is Gaussian noise with $\sigma = 1.0$, making the task more challenging.

S3 Experiments on CIFAR-10

Following [7], we construct a Cluttered CIFAR-10 classification task. Each input is a $3 \times 32 \times 64$ composite image formed by concatenating a 32×32 CIFAR-10 target image (left half) with a 32×32 distractor (right half). For easy samples, the distractor is a black (all-zeros) image. For *hard* samples, the distractor is drawn from a Gaussian distribution $\mathcal{N}(0, \sigma^2)$ applied independently to each pixel and channel, with $\sigma = 1.0$. All 10 CIFAR-10 classes are used, so that the random-chance test error is 10%. Figure S1 shows example composite images.

Motivated by the optimal curriculum derived in the main text, we parameterize the Easy-Hard-Easy training schedule with a single parameter $a \in [0, 50]$, expressed as a percentage of the total training set. Training proceeds in three phases:

1. **Initial easy phase**: the first a% of training samples are easy;
2. **Hard phase**: the next 50% are hard;
3. **Final easy phase**: the remaining $(50 - a)$% are easy.

Every schedule presents exactly 50% easy and 50% hard samples, only the presentation order differs. At the extremes, $a = 0$ corresponds to the Anti-Curriculum strategy, and $a = 50$ corresponds to the standard Curriculum strategy.

The $N = 50{,}000$ CIFAR-10 training images are randomly partitioned into 25,000 easy and 25,000 hard samples. This assignment is held constant across all values of a, so that every schedule trains on the *same* set of easy and hard images—only the ordering changes. Each image always receives the same noise realization across all experimental conditions. Within the easy and hard groups, samples are shuffled with a seed that varies per repetition, so that different runs see different within-group orderings while maintaining the same phase structure. The test set consists of the 10,000 CIFAR-10 test images, constructed as a fixed 50/50 easy/hard mixture.

We use a simple convolutional neural network with three convolutional layers:

- Conv2d($3 \to 32$, 3×3, padding$= 1$) $\to$ ReLU $\to$ MaxPool(2×2)
- Conv2d($32 \to 64$, 3×3, padding$= 1$) $\to$ ReLU $\to$ MaxPool(2×2)
- Conv2d($64 \to 64$, 3×3, padding$= 1$) $\to$ ReLU $\to$ MaxPool(2×2)

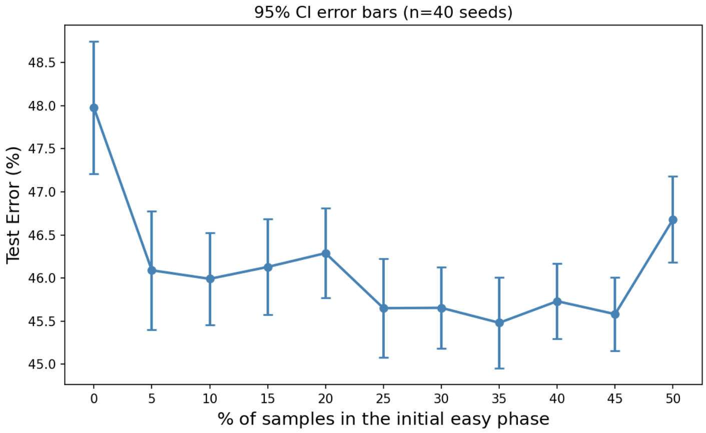

Figure S2: Test error (mean over 30 model initialization seeds, with error bar corresponding to 95% confidence intervals) as a function of the initial easy fraction a on Cluttered CIFAR-10. The extremes $a = 0$ (Anti-Curriculum) and $a = 50$ (Curriculum) yield the highest error, while intermediate values achieve the lowest error, confirming the advantage of the Easy-Hard-Easy strategy. Random-chance error is 90%.

followed by a fully connected classifier: Flatten $\to$ Linear($64 \times 4 \times 8 \to 256$) $\to$ ReLU $\to$ Linear($256 \to 10$). The input dimensionality is $3 \times 32 \times 64$ to accommodate the composite images. Training is performed online: each training sample is presented exactly once, in mini-batches of size 16. We use the Adam optimizer with a constant learning rate of 10^{-3} and cross-entropy loss. No learning rate scheduler is employed.

For each value of a, we construct the corresponding easy-hard-easy training sequence and train the model from scratch (single online pass). To quantify variability due to model initialization, we repeat each run 30 times with distinct weight initialization seeds, while keeping the training sequence identical across repetitions. In Figure S2, we show the mean test error as a function of a. Interestingly, we find that the easy-hard-easy, originally derived in the main text for our minimal model, with $a = 35\%$ outperforms both Curriculum ($a = 50\%$) and Anti-Curriculum ($a = 0\%$).

S4 Supplementary figures and additional details

The initial conditions for the order parameters used in Fig. 3 of the main text are

$$R = \frac{\boldsymbol{w}^\top \boldsymbol{\mu_c}}{N} = \begin{pmatrix} 0.116 & 0.029 \\ -0.005 & 0.104 \end{pmatrix}, \qquad Q = \frac{\boldsymbol{w}^\top \boldsymbol{w}}{N} = \begin{pmatrix} 0.25 & 0.003 \\ 0.003 & 0.25 \end{pmatrix},$$
$$\Omega_{(1,1)(1,1)} = \frac{\boldsymbol{\mu}_{1,1} \cdot \boldsymbol{\mu}_{1,1}}{N} = 0.947\,, \qquad \Omega_{(1,2)(1,2)} = \frac{\boldsymbol{\mu}_{1,2} \cdot \boldsymbol{\mu}_{1,2}}{N} = 0.990\,. \tag{S71}$$

The initial conditions for the order parameters used in Fig. 4 are

$$R = \frac{\boldsymbol{w}^\top \boldsymbol{\mu_c}}{N} = \begin{pmatrix} 0.339 & 0.200 \\ 0.173 & 0.263 \end{pmatrix}, \qquad Q = \frac{\boldsymbol{w}^\top \boldsymbol{w}}{N} = \begin{pmatrix} 1 & 0.00068 \\ 0.00068 & 1 \end{pmatrix},$$
$$\Omega_{(1,1)(1,1)} = \frac{\boldsymbol{\mu}_{1,1} \cdot \boldsymbol{\mu}_{1,1}}{N} = 1.737\,, \qquad \Omega_{(1,2)(1,2)} = \frac{\boldsymbol{\mu}_{1,2} \cdot \boldsymbol{\mu}_{1,2}}{N} = 1.158\,. \tag{S72}$$

The test set used in Fig. 4b contains 13996 examples. The standard deviations of the clusters are $\sigma_{1,1} = 0.05$ and $\sigma_{1,2} = 0.033$. The cluster membership probability is $p_c([c_1 = 1, c_2 = 1]) = 0.47$ and $p_c([c_1 = 2, c_2 = 1]) = 0.53$. The initial conditions for the order parameters used in Fig. S6

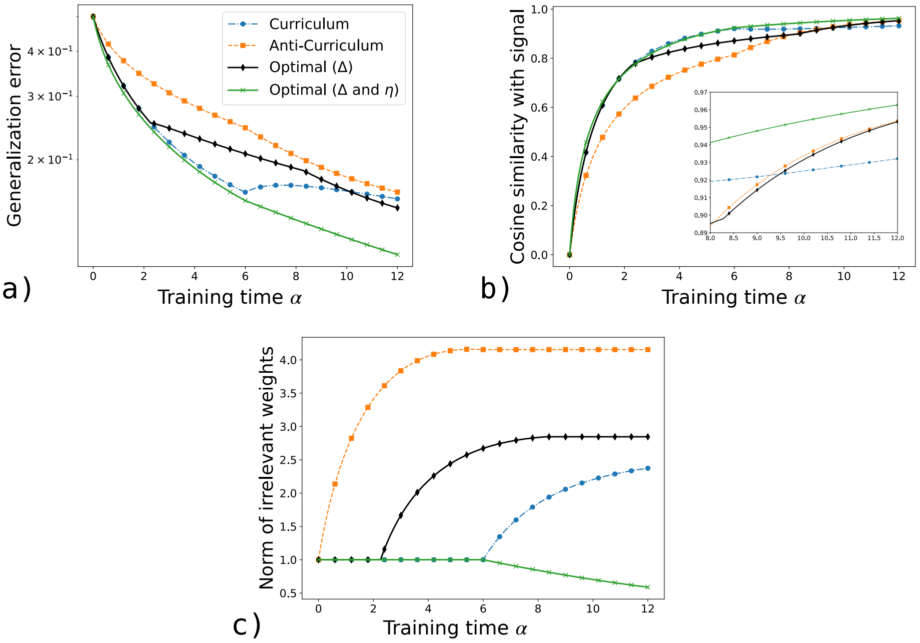

Figure S3: Dynamics of the curriculum learning problem under different training schedules—curriculum (easy to hard) at $\eta = 3$, anti-curriculum (hard to easy) at $\eta = 3$, the optimal difficulty protocol at $\eta = 3$, and the optimal protocol obtained by jointly optimizing Δ and η. **(a)** Generalization error vs. normalized training time $\alpha = \mu/N$. **(b)** Cosine similarity $M_{11}/\sqrt{TQ_{11}}$ with the target signal (inset zooms into the late-training regime). **(c)** Squared norm of irrelevant weights Q_{22} vs. α. **Parameters:** $\alpha_F = 12$, $\Delta_1 = 0$, $\Delta_2 = 2$, $\eta = 3$, $\lambda = 0$, $T = 2$. **Initial conditions:** $Q_{11} = Q_{22} = 1$, $M_{11} = 0$.

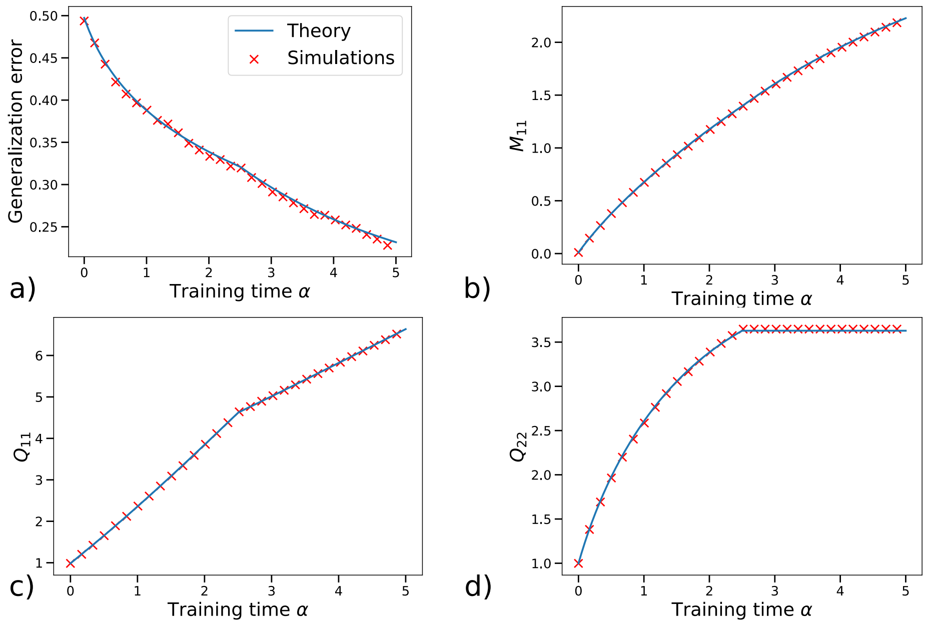

Figure S4: Comparison between theory and simulations in the curriculum learning problem: **a)** generalization error, **b)** teacher-student overlap M_{11}, **c)** squared norm Q_{11} of the relevant weights, and **d)** squared norm Q_{22} of the irrelevant weights. The continuous blue lines have been obtained by integrating numerically the ODEs in Eqs. (S22), while the red crosses are the results of numerical simulations of a single trajectory with $N = 30000$. The protocol is anti-curriculum with equal proportions of easy and hard samples. **Parameters:** $\alpha_F = 5$, $\lambda = 0$, $\eta = 3$, $\Delta_1 = 0$, $\Delta_2 = 2$, $T_{11} = 1$. **Initial conditions:** $Q_{11} = 0.984$**,** $Q_{22} = 0.998$**,** $M_{11} = 0.01$**.**

are

$$R = \frac{\boldsymbol{w}^\top \boldsymbol{\mu_c}}{N} = \begin{pmatrix} 0.099 & -0.005 \\ -0.002 & 0.102 \end{pmatrix}, \qquad Q = \frac{\boldsymbol{w}^\top \boldsymbol{w}}{N} = \begin{pmatrix} 0.25 & -0.002 \\ -0.002 & 0.25 \end{pmatrix},$$
$$\Omega_{(1,1)(1,1)} = \frac{\boldsymbol{\mu}_{1,1} \cdot \boldsymbol{\mu}_{1,1}}{N} = 0.976\,, \qquad \Omega_{(1,2)(1,2)} = \frac{\boldsymbol{\mu}_{1,2} \cdot \boldsymbol{\mu}_{1,2}}{N} = 1.014\,. \tag{S73}$$

These initial conditions are obtained by either randomly generating the centroids $\boldsymbol{\mu}_{1,1}$ and $\boldsymbol{\mu}_{1,2}$ or by fitting them to the data (MNIST datasets). The student parameters $\boldsymbol{w}$ are also randomly generated with a finite overlap with the centroids.

S5 Numerical simulations

In this section, we validate our theoretical predictions against numerical simulations for the three scenarios studied: curriculum learning (Fig. S4), dropout regularization (Fig. S5), and denoising autoencoders (Fig. S6). For each case, the theoretical curves are obtained by numerically integrating the respective ODEs, obtained in the high-dimensional limit $N \to \infty$. The simulations are instead obtained for a single SGD trajectory at large but finite N. We observe good agreement between theory and simulations. The code for reproducing the numerical simulations is available at [3].

References

[1] J. A. Andersson, J. Gillis, G. Horn, J. B. Rawlings, and M. Diehl. Casadi: a software framework for nonlinear optimization and optimal control. *Mathematical Programming Computation*, 11:1–36, 2019.

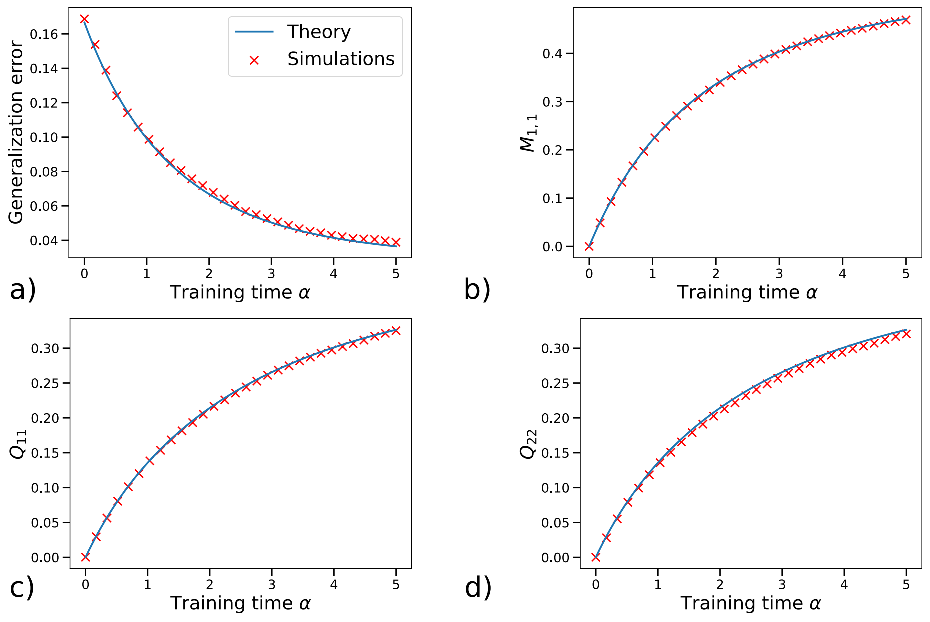

Figure S5: Comparison between theory and simulations for dropout regularization: **a)** generalization error, **b)** teacher-student overlap $M_{1,1}$, **c)** squared norm Q_{11}, and **d)** squared norm Q_{22}. The continuous blue lines have been obtained by integrating numerically the ODEs in Eqs. (S26-S27), while the red crosses are the results of numerical simulations of a single trajectory with $N = 30000$. **Parameters:** $\alpha_F = 5$, $\eta = 1$, $\sigma_n = 0.3$, $p(\alpha) = p_f = 0.7$, $T_{11} = 1$. **Initial conditions:** $Q_{ij} = M_{nk} = 0$.

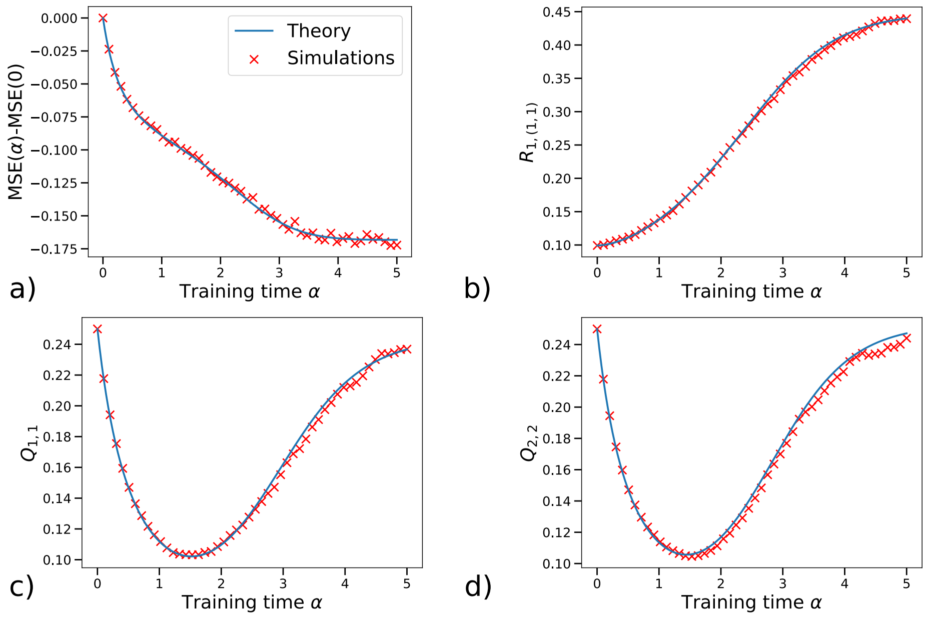

Figure S6: Comparison between theory and simulations for the denoising autoencoder model: **a)** mean square error improvement, **b)** student-centroid overlap $R_{1,(1,1)}$, **c)** squared norm Q_{11}. The continuous blue lines have been obtained by integrating numerically the ODEs in Eqs. (S54) and (S61), while the red crosses are the results of numerical simulations of a single trajectory with $N = 10000$. **Parameters:** $\alpha_F = 1$, $\eta = 2$, $B(\alpha) = \bar{B} = 5$, $K = C_1 = 2$, $\sigma = 0.1$, $g(z) = z$, $\Delta(\alpha) = \Delta_F = 0.8$. The skip connection b is fixed ($\eta_b = 0$) to the optimal value b^*. Initial conditions are given in (S73).

[2] S. Goldt, M. Advani, A. M. Saxe, F. Krzakala, and L. Zdeborová. Dynamics of stochastic gradient descent for two-layer neural networks in the teacher-student setup. *Advances in neural information processing systems*, 32, 2019.

[3] F. Mignacco and F. Mori. Github repository. https://github.com/francescomori/optimal_learning/, 2025.

[4] F. Mori and F. Mignacco. Analytic theory of dropout regularization. *Phys. Rev. E*, 112: 045301, 2025.

[5] F. Mori, S. S. Mannelli, and F. Mignacco. Optimal protocols for continual learning via statistical physics and control theory. In *International Conference on Learning Representations (ICLR)*, 2025. URL https://openreview.net/forum?id=rhhQjGj09A.

[6] L. Pontryagin. Some mathematical problems arising in connection with the theory of optimal automatic control systems. In *Proc. Conf. on Basic Problems in Automatic Control and Regulation*, 1957.

[7] L. Saglietti, S. Mannelli, and A. Saxe. An analytical theory of curriculum learning in teacher-student networks. *Advances in Neural Information Processing Systems*, 35:21113–21127, 2022.
